# Multifunctional Poly(thioctic acid) Composite Hydrogels with Self-Healing, Antibacterial, Antioxidant, and Adhesive Properties

**DOI:** 10.3390/ma19132695

**Published:** 2026-06-23

**Authors:** Yang Yuan, Jiawei Zhang, Fangzheng Yu, Chen Wang, Jiale He, Zheng Zhao

**Affiliations:** 1 Sanya Science and Education Innovation Park, Wuhan University of Technology, Sanya 572000, China; 348318@whut.edu.cn (Y.Y.); zjw9801@126.com (J.Z.); 348317@whut.edu.cn (F.Y.); wangchen__123@163.com (C.W.); hjl2024305118@163.com (J.H.); 2 State Key Laboratory of Advanced Technology for Materials Synthesis and Processing, Wuhan University of Technology, Wuhan 430070, China

**Keywords:** thioctic acid, salicylic acid, adhesive, antioxidant, antibacterial

## Abstract

Bacterial infections and excessive reactive oxygen species (ROS) severely impede wound healing. However, traditional hydrogels often lack the integrated antibacterial and antioxidant properties required for effective treatment. To overcome these limitations, a natural thioctic acid (TA)-based multifunctional composite hydrogel (PTA-Arg/SAS) was developed. Arginine (Arg) served as a green inducer for the aqueous ring-opening polymerization of TA. Concurrently, salicylic acid-grafted sericin (SAS) was introduced to inhibit poly(thioctic acid) (PTA) depolymerization via the formation of stable sulfur-aryl (S-Ar) bonds. The hydrogel exhibits self-healing capability, injectability, and robust tissue adhesion to porcine skin (1877 Pa dry; 1663 Pa wet). Furthermore, SAS endowed the system with potent antibacterial (99.1% against *E. coli*, 97% against *S. aureus*) and antioxidant activities (98.2% ABTS and 72.7% DPPH radical scavenging rates). In vitro evaluations confirmed the viability of L929 cells (>98% over 3 days) and a negligible hemolysis ratio (<5%). Consequently, this study provides a strategy for fabricating next-generation bioactive dressings for complex wound management.

## 1. Introduction

Wound healing is a complex physiological process involving multicellular interactions [1]. Severe bacterial infections and excessive inflammation can continuously deteriorate the local microenvironment, hindering normal cell proliferation and differentiation, and ultimately stalling the healing process [2]. Hydrogels are widely regarded as ideal wound dressings as they can maintain a moist microenvironment and provide a physical barrier [3]. However, traditional hydrogels often serve merely as passive barriers, lacking intrinsic antibacterial and antioxidant properties [4,5]. Furthermore, they frequently exhibit inadequate tissue adhesiveness, making them prone to detachment during dynamic skin stretching and deformation [6,7]. The failure of this physical barrier not only increases the risk of secondary bacterial invasion but also necessitates frequent dressing changes, which can damage newly formed tissues [8,9]. Therefore, it is necessary to develop a multifunctional hydrogel dressing with antibacterial, antioxidant, and tissue-adhesive properties.

Thioctic acid (TA) is a natural coenzyme widely distributed in plants and animals and possesses favorable biocompatibility and antioxidant activity [10]. A TA molecule contains a five-membered cyclic disulfide bond, which is prone to undergo ring-opening polymerization to form poly(thioctic acid) (PTA) under thermal or alkaline induction [11,12]. Benefiting from abundant dynamic disulfide bonds and free carboxyl groups within the PTA network, PTA-based hydrogels exhibit good self-healing and tissue-adhesive properties [13]. However, the poor aqueous solubility of TA remains a major obstacle to the fabrication of hydrogels. Traditional dissolution strategies often rely on toxic solvents [14,15]. For instance, Fang et al. reported the in situ polymerization of TA in halometallate ionic liquids for ionogel fabrication [16]. In addition, Feng et al. fabricated a TA-based conductive hydrogel for flexible electronic sensing, employing 30% aqueous KOH solution as the polymerization medium [17]. To ensure optimal biocompatibility for wound management, the processing of TA-based dressings must circumvent irritating or corrosive media. Arginine (Arg), a conditionally essential basic amino acid, not only possesses broad-spectrum antibacterial activity but also serves as a safe and mild biological inducer [18]. It can effectively dissolve TA in a pure aqueous phase and achieve its controllable polymerization. Nevertheless, the resulting aqueous PTA network is intrinsically metastable and highly susceptible to depolymerization [19].

Phenolic compounds can be coupled with free sulfur radicals at the terminals of PTA to form thermodynamically stable sulfur-aryl bonds (S-Ar bonds), thereby effectively anchoring the PTA network and inhibiting its depolymerization. As a typical natural phenolic molecule, salicylic acid (SA) can provide anchoring sites for S-Ar bonds [20]. However, free SA features poor aqueous solubility, susceptibility to oxidative deactivation, and burst release, which limit its practical applications [21,22]. To overcome these limitations, we introduced natural sericin (Ser), a hydrophilic biomacromolecule renowned for its favorable mechanical and antioxidant properties [23,24]. By adopting a macromolecular grafting strategy, SA was covalently bonded onto the Ser backbone to prepare SAS. This design effectively resolves the drawbacks of poor aqueous solubility and burst release of small-molecular SA. More importantly, the free phenolic hydroxyl groups on SAS act as macromolecular crosslinkers that effectively anchor the dynamic PTA network via S–Ar bonds. This strategy stabilizes the dynamic network of PTA and ensures sustained anti-inflammatory and antibacterial efficacy.

**Figure 1:**
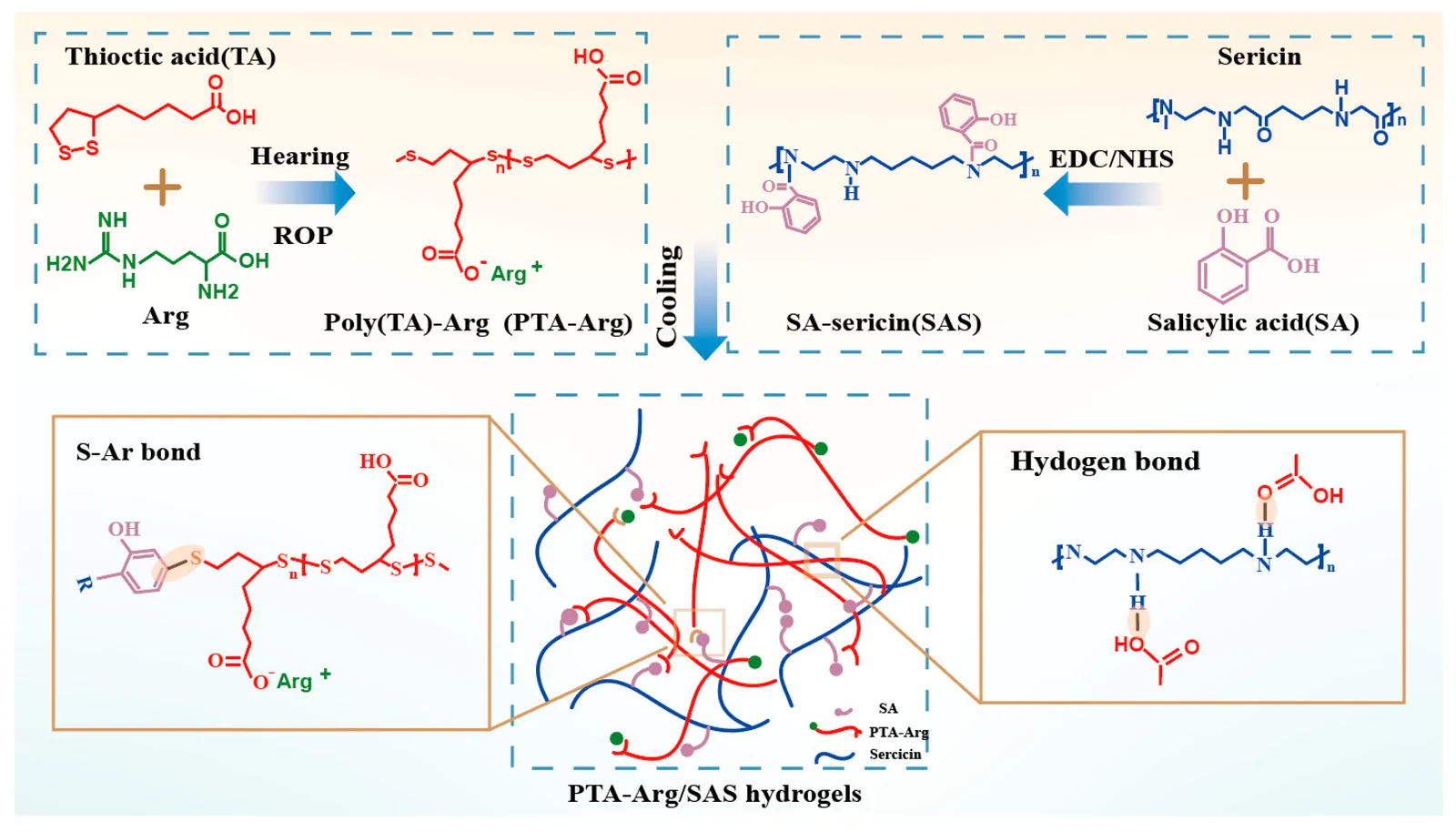
The preparation of PTA-Arg/SAS hydrogel.

In this study, a multifunctional composite hydrogel (PTA-Arg/SAS) was prepared by integrating an Arg-induced PTA network with SAS (Figure 1). To reduce the reliance on irritating media often employed in conventional processes, Arg served as a relatively mild inducer for the aqueous ring-opening polymerization of TA. Additionally, to address the tendency of the aqueous PTA network to depolymerize, as well as the fast release profile of free SA, SAS was incorporated. The free phenolic hydroxyl groups on SAS may act as crosslinking sites, contributing to the stabilization of the dynamic PTA network via S-Ar bonds. This approach is beneficial for restraining the depolymerization of PTA and concurrently endowing the hydrogel with the functional features of SAS. Furthermore, the physical and chemical properties, wet adhesion, antioxidant and antibacterial abilities, and biocompatibility of the PTA-Arg/SAS hydrogels were systematically investigated.

## 2. Materials and Methods

### 2.1. Materials

Thioctic acid, salicylic acid (99.5%), ethanol (99.5%), sericin, n-hexane, 1-(3-Dimethylaminopropyl)-3-ethylcarbodiimide hydrochloride (EDC, 99%), N-Hydroxy succinimide (NHS, 98%), 1,1-diphenyl-2-trinitrophenylhydrazine (DPPH), and 2,2′-azino-bis-3-ethylbenzthiazoline-6-sulphonic acid (ABTS) were all purchased from Aladdin (Shanghai, China). Gibco Life Sciences (Grand Island, NY, USA) supplied the RPMI 1640 medium. All other necessary reagents, including fetal bovine serum (FBS), penicillin–streptomycin (PS), calcium yellow-green AM stain, Cell Counting Kit-8 (CCK-8), and phosphate-buffered saline (PBS), were sourced directly from Beyotime Biotechnology (Shanghai, China).

### 2.2. Preparation of SAS

We reacted SA (1 g) with EDC (1.39 g) and NHS (0.834 g) in 50 mL of ethanol at pH 5.5 for 40 min. Then, we mixed this with an aqueous Ser solution (2 g, 100 mL) and allowed it to react for 6 h. Finally, SAS powder was obtained after dialysis and freeze-drying.

### 2.3. Preparation of PTA-Arg/SAS Hydrogel

We dissolved TA (1.2 g) in an Arg solution (0.6 g, 10 mL) at 70 °C for 20 min. Then, we added SAS powder (1, 3, and 5 wt%) and stirred for 1 h while maintaining the solution at pH 7. Finally, we molded the mixture and allowed it to cool to room temperature to form PTA-Arg/SAS hydrogels.

### 2.4. Characterization of SAS Powder and PTA-Arg/SAS Hydrogel

To characterize the hydrogel structure, we used Fourier transform infrared (FTIR) spectroscopy (Nexus, Thermo Nicolet, Madison, WI, USA) and Raman spectroscopy (MicroTEQ-S1, Ocean Optics, Shanghai, China) to confirm the grafting of SA onto Ser. The FTIR spectra were recorded in the range of 600–3800 cm^−1^. Ultraviolet–visible (UV–vis) absorption spectra were obtained using a spectrophotometer (UV-2550, Shimadzu, Kyoto, Japan) in the range of 280–500 nm using tetrahydrofuran as the solvent (100 μL, 200g/L).

### 2.5. Ultraviolet Protection Performance Test

Rectangular hydrogel samples (20 × 20 × 1 mm^3^) were prepared. We recorded the optical transmittance of the hydrogels from 200 to 800 nm using a UV–Vis spectrophotometer.

### 2.6. Morphological and Structural Characterization

The microstructure and morphology of the hydrogels were observed using a scanning electron microscope (JSM-IT200, Jeol, Tokyo, Japan). First, the hydrogel samples were freeze-dried and sputter-coated with gold, followed by surface scanning. Subsequently, the pore size and distribution of the hydrogels were analyzed from representative SEM images using ImageJ software (ImageJ 1.54f). For each sample, 50 randomly selected pores were measured to calculate the average pore size and generate the pore size distribution histograms.

### 2.7. Porosity Test

A solvent displacement technique was employed to assess hydrogel porosity. First, the dry weight (M_a_) and geometric volume (V, cm^3^) of the freeze-dried cylinders were determined. The samples were subsequently submerged in n-hexane until equilibrium saturation was reached. Following the gentle removal of surface-adhering n-hexane, the wet weight (M_b_) was recorded. The equation is as follows:

(1) $$Porosity=\frac{M_{b}-M_{a}}{V\times \rho }$$

where ρ is 0.6603 g/cm^3^.

### 2.8. Moisture Retention Performance Test

Rectangular hydrogel samples (20 × 20 × 5 mm^3^) with different SAS contents were prepared, and their initial masses (M_0_) were recorded. We incubated the samples at 37 °C and 65% RH and weighed them at predetermined time intervals until a constant mass (M) was reached. The equation is as follows:

(2) $$Water\;retention(\%)=\frac{M}{M_{0}}$$

### 2.9. Water Content and Swelling Ratio Test

We recorded the hydrogel mass as M_c_. The hydrogel was then freeze-dried, and the dry mass was recorded as M_e_. The equation is as follows:

(3) $$Water\;content(\%)=\frac{M_{c}-M_{e}}{M_{c}}\times 100$$

We swelled the freeze-dried hydrogels in deionized water at 37 °C for 40 h to reach equilibrium and then recorded the swollen mass as M_f_. The equation is as follows:

(4) $$Swelling\;ratio(\%)=\frac{M_{f}}{M_{e}}$$

### 2.10. Degradation Test

We evaluated the degradation of the hydrogels by soaking them in PBS. Specifically, we completely submerged 500 mg of the dried hydrogel into 50 mL of PBS and placed it into a shaking incubator at 37 °C and 100 rpm. We removed the samples at scheduled time points, dried them, and weighed them to track the mass changes. The equation is as follows:

(5) $$Degradation\;rate(\%)=\frac{{500-W}_{S}}{500}\times 100$$

where W_s_ is the weight of the dried hydrogel at the specific time point.

### 2.11. Rheological Test

We evaluated the rheological behavior of the hydrogels using a rotational rheometer (Kinexus pro^+^, Netzsch, Bavaria, Germany). For all tests, we used a 20 mm parallel plate geometry with a 1000 μm gap, and the temperature was maintained at 37 °C. Prior to measurement, each sample was allowed to equilibrate for 3 min to relieve any loading-induced residual stresses. First, we performed strain amplitude sweeps (0.1–4000% strain) at a fixed frequency of 1 rad/s to determine the linear viscoelasticity region, stability, and critical strain points. Additionally, we performed alternating step-strain tests between 1% and 2000% strain. Each strain interval was maintained for 120 s at a fixed frequency of 1 Hz, and the alternating cycle was repeated 4 times. The self-healing efficiency of the hydrogel was quantitatively estimated based on the step-strain rheological measurements. It was calculated as the ratio of the recovered storage modulus after each high-strain damage cycle (G′_H_) to the initial baseline storage modulus (G′_I_). The equation is as follows:

(6) $$\mathrm{Healing}\;\mathrm{efficiency}\;(\%)=\frac{{G^{\prime }}_{H}}{{G^{\prime }}_{I}}$$

### 2.12. Self-Healing Performance Test

Two semicircular PTA-Arg/SAS5 hydrogel blocks with different colors were prepared. The two hydrogels were closely attached to each other at room temperature. We observed the interfacial fusion to assess self-healing.

### 2.13. Adhesion Performance Test

The adhesive strength of hydrogels was evaluated by a lap-shear test. The sample was prepared by cutting pieces of pork skin to 50 mm × 25 mm and laminating two pieces together with the PTA-Arg/SAS hydrogel (adhesive area: 40 mm × 25 mm). Both dry pork skin surfaces and PBS-wetted pork skin surfaces were used for testing. We stretched the samples at a rate of 50 mm/min. The adhesion test on metal sheet surfaces followed the same procedure. The equation is as follows:

(7) $$\mathrm{Adhesion}\;\mathrm{strength}\;(\mathrm{kpa})=\frac{F_{p}}{S}$$

where F_p_ is the maximum load force, and S is the bonding area.

Additionally, the macroscopic adhesion capability to various materials was evaluated using metal, plastic, and foam substrates. The PTA-Arg/SAS5 hydrogel was evenly coated on the elbow and finger joints and then bent to test the fitting performance of the hydrogel.

### 2.14. Injectable Performance Test

The hydrogel was continuously written into a “WUT” pattern on a Petri dish through a 21G needle to assess its injectability. Additionally, a quantitative injection test was conducted using a universal testing machine (CMT6503, MTS, Eden Prairie, MN, USA) equipped with a 50 N load cell at a uniform extrusion speed of 50 mm/min. The recorded peak force during this process was defined as the injection thrust [25].

### 2.15. Antioxidant Activity Test

The ABTS solution was prepared by first dissolving 0.1015 g of ABTS powder in 25 mL of ethanol. Separately, 0.0148 g of ammonium persulfate was dissolved in another 25 mL of ethanol. These two solutions were then mixed and allowed to stand overnight to generate the active ABTS reagent. The resulting solution was diluted with ethanol to an absorbance of 0.70 ± 0.02 at 734 nm before use. Subsequently, 100 μL of the sample or deionized water (blank control) was added to 1 mL of the prepared ABTS reagent. After incubation at 37 °C for 15 min in the dark, the absorbance was measured at 734 nm. The equation is as follows:

(8) $$\mathrm{ABTS}\;\mathrm{scavenging}(\%)=\frac{A_{m}-A_{n}}{A_{m}}\times 100$$

where A_m_ and A_n_ represent the absorbance values of the deionized water group and the test sample group, respectively.

A 0.04 mmol/L DPPH-ethanol solution was prepared. The procedure of the DPPH test was similar, with the reaction kept away from light for 30 min. We measured the absorbance at 517 nm. The equation is as follows:

(9) $$\mathrm{DPPH}\;\mathrm{scavenging}(\%)=\frac{A_{o}-A_{p}}{A_{o}}$$

where A_o_ and A_p_ are the absorbance values of the deionized water group and the test sample group, respectively.

### 2.16. Antibacterial Test

We tested the hydrogels against *E. coli* and *S. aureus* via agar plating [26]. First, we placed 100 μL of the hydrogel samples into 48-well plates and UV-sterilized them for 12 h. We then added 1 mL of bacterial suspension (roughly 1 × 10^5^ CFU/mL) to each well. Including the blank controls, we incubated the plates at 37 °C with 5% CO_2_ and 80% RH on a shaker (120 rpm) for 12 h. After this, we serially diluted the mixtures by 10^5^-fold with sterile PBS. Subsequently, 100 μL of the diluted solution was spread onto LB agar plates. After incubation at 37 °C for 14 h, the plates were photographed, and colony counts were performed. The equation is as follows:

(10) $$\mathrm{Antibacterial}\;\mathrm{rate}(\%)=\left(\frac{{A_{q}-A}_{r}}{A_{q}}\right)\times 100$$

where A_q_ and A_r_ are the bacterial colony counts of the blank group and the test sample group, respectively.

### 2.17. Biocompatibility Test

To evaluate the in vitro cytocompatibility of our hydrogels, we performed a CCK-8 assay using L929 fibroblasts. Initially, we seeded L929 cells (Wuhan Servicebio Technology Co., Ltd., Wuhan, China) into 96-well plates at a density of 5000 cells/well and incubated them at 37 °C in a 5% CO2 atmosphere using complete RPMI 1640 medium (supplemented with 10% FBS and 1% penicillin/streptomycin). Concurrently, we prepared hydrogel extracts by soaking the materials in the same complete medium for 24 h and passing the resulting liquid through a sterile syringe filter. Once the cells had attached for 24 h, we discarded the original medium and introduced the PTA/AG hydrogel extracts. After co-culturing the cells for 1, 2, and 3 days, we removed the extract solutions and added 100 µL of CCK-8 working reagent to each well. Finally, we incubated the plates for another 2 h under standard conditions and measured the optical density (OD) at 450 nm with a microplate reader. The equation is as follows:

(11) $$\mathrm{Cell}\;\mathrm{viability}(\%)=\frac{A_{1}}{A_{0}}\times 100$$

where A_0_ and A_1_ are the absorbance values of the blank group and the test sample group, respectively.

For a more intuitive assessment of cell morphology and hydrogel biocompatibility, we performed a live/dead viability assay on the co-cultured cells. After introducing the Calcein-AM/PI mixed staining solution, we kept the samples protected from light at 37 °C for a 30 min period. Finally, the cellular fluorescence states were recorded via fluorescence microscopy.

### 2.18. Blood Compatibility Test

To assess the hemocompatibility of hydrogels, a hemolysis assay was performed using commercially available Sprague–Dawley rat erythrocytes (Oumarsi, Shanghai, China). Briefly, the purchased erythrocytes were purified via PBS washing to yield a 4% (*v*/*v*) erythrocyte suspension. For the assay, 100 μL of the hydrogel was mixed with 2 mL of the cellular suspension and maintained at 37 °C for one hour. Concurrent tests using pure water and PBS served as positive and negative controls, respectively. Following the incubation period, the mixtures underwent centrifugation for 10 min. The optical densities of the resulting supernatants were then measured at 545 nm. The equation is as follows:

(12) $$\mathrm{Hemolysis}\;\mathrm{rate}=\frac{A_{d}-A_{f}}{A_{e}-A_{f}}$$

where A_d_, A_e_, and A_f_ represent the absorbance values after treatment with pure water, PBS, and hydrogel, respectively.

### 2.19. Statistical Analysis

More than three independent samples were used in each group of experiments. Significance analysis was performed using IBM SPSS Statistics 27 software (* *p* < 0.05, ** *p* < 0.01, *** *p* < 0.001).

## 3. Results

### 3.1. Synthesis and Characterization of PTA-Arg/SAS

**Figure 2:**
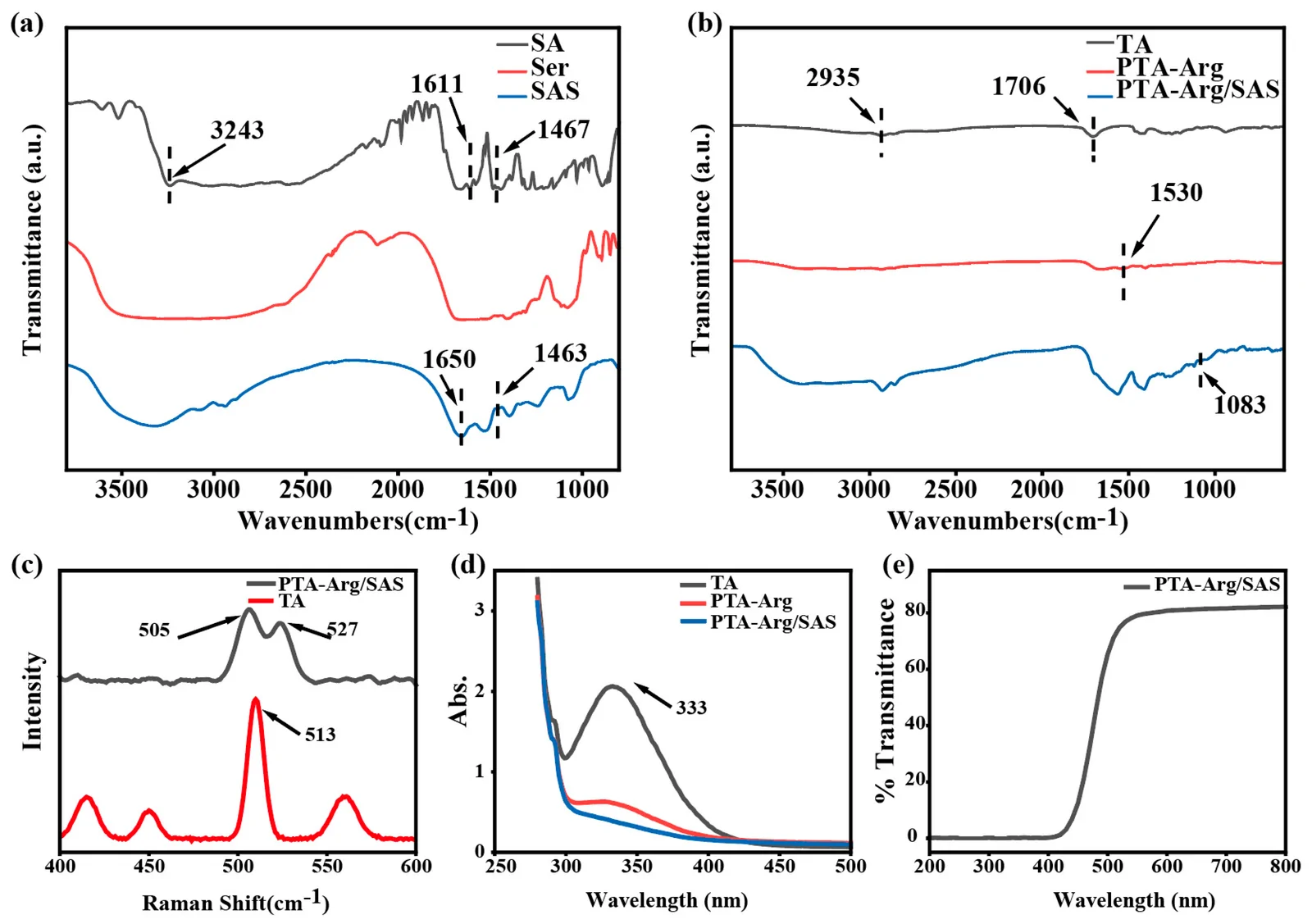
(**a**,**b**) FTIR spectra of SAS and PTA-Arg/SAS. (**c**) Raman of TA and PTA-Arg/SAS. (**d**) UV–vis absorption of hydrogels. (**e**) Transmittance of PTA-Arg/SAS.

Figure 2a displays a broad O-H stretching band at 3243 cm^−1^ [27,28]. The spectra also exhibit carboxyl C=O (1611 cm^−1^) and aromatic C=C (1467 cm^−1^) skeletal vibrations [29,30]. Amide bond formation between the carboxyl groups of SA and amine groups of Ser generated a new absorption peak at 1650 cm^−1^ in the SAS spectrum [31]. Additionally, the SA aromatic ring exhibited a skeletal vibration at 1463 cm^−1^ [32]. In Figure 2b, pure TA displays free carboxyl C=O stretching (1706 cm^−1^) and aliphatic C-H vibrations at 2935 cm^−1^ [16,33]. In the PTA-Arg spectrum, a peak at 1530 cm^−1^ was assigned to the characteristic absorption of -COO^−^ resulting from the partial deprotonation of TA induced by the alkaline Arg solution [34]. The peak at 1083 cm^−1^ was ascribed to the S-Ar bonds, verifying the role of SAS as a sulfur radical scavenger [35]. Raman spectra further confirmed that the incorporation of SAS could inhibit the depolymerization of PTA. As shown in Figure 2c, the S-S stretching peak of the pure TA monomer at 510 cm^−1^ split and shifted to 505 and 527 cm^−1^ upon the formation of the composite hydrogel, confirming the generation of S-Ar bonds [35,36]. UV–vis absorption spectra further confirmed the formation of the PTA network. Pure TA displayed a characteristic 333 nm absorption band, representing the five-membered cyclic disulfide structure (Figure 2d) [37]. In contrast, the intensity of the corresponding peak in the PTA-Arg and PTA-Arg/SAS hydrogels was diminished. This spectral variation suggested that the cyclic structure of TA underwent ring-opening polymerization to form linear PTA chains [38].

### 3.2. UV Protection Performance of PTA-Arg/SAS

The favorable transparency of the hydrogel benefited both tissue protection and non-invasive in situ monitoring. Figure 2e shows that the PTA-Arg/SAS hydrogel effectively blocks UV radiation in the 200–400 nm region. The aromatic-rich moieties of SAS were primarily responsible for this UV-shielding effect [39,40]. In the visible region of 400–800 nm, the hydrogel retained high optical transmittance above 80%. Therefore, the prepared hydrogel possessed both effective UV-shielding capacity and high transparency, enabling the material to block harmful ultraviolet radiation while allowing for real-time visual observation of the underlying tissue.

### 3.3. Morphology and Porosity of PTA-Arg/SAS

**Figure 3:**
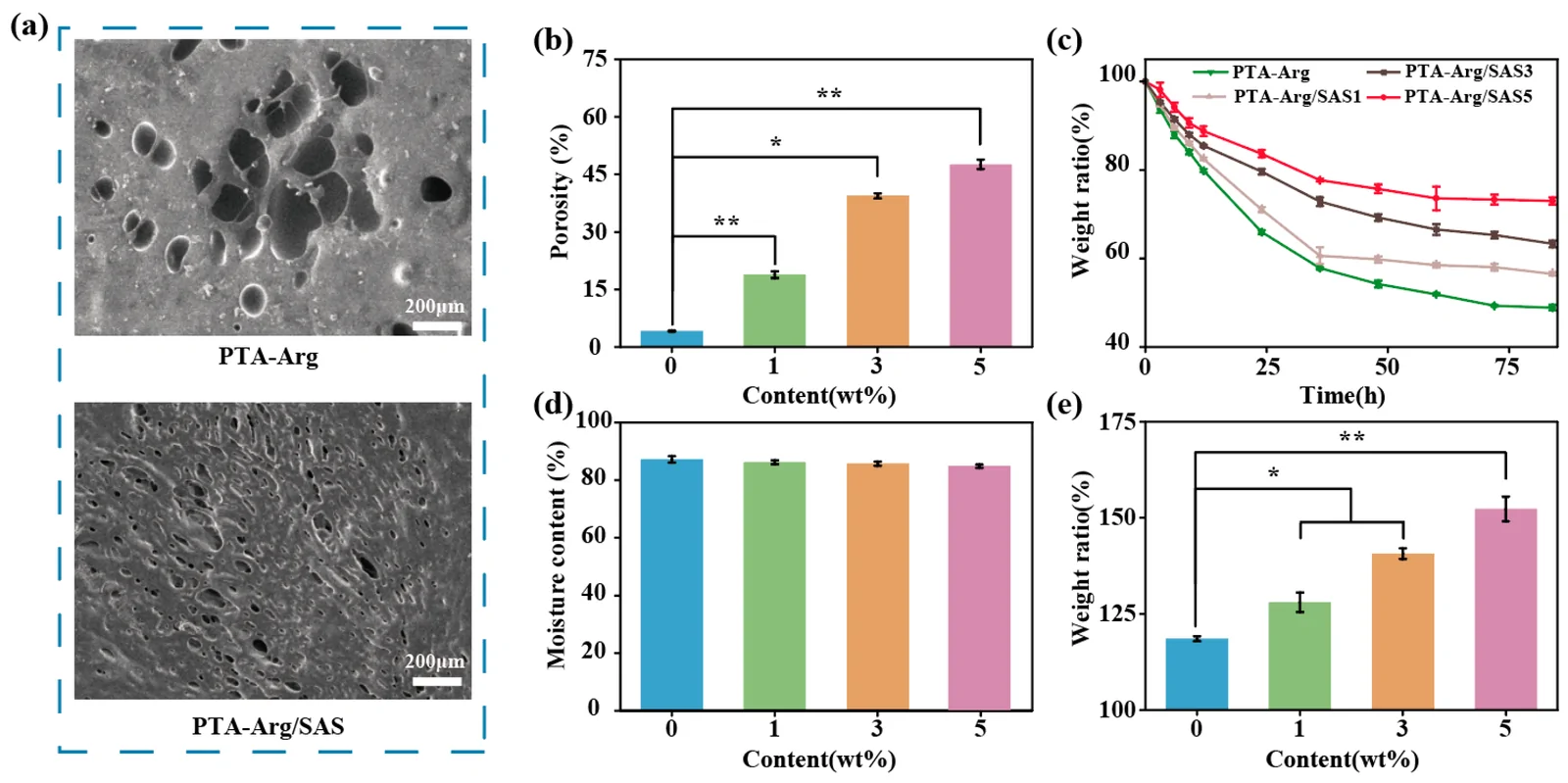
(**a**) SEM structural characterization of PTA-Arg/SAS. (**b**) Porosity of PTA-Arg/SAS (n = 3, ** *p* < 0.01 and * *p* < 0.05). (**c**) Water retention capacity of PTA-Arg/SAS. (**d**) Water content of PTA-Arg/SAS. (**e**) Swelling property of PTA-Arg/SAS (n = 3, ** *p* < 0.01 and * *p* < 0.05).

**Figure A1:**
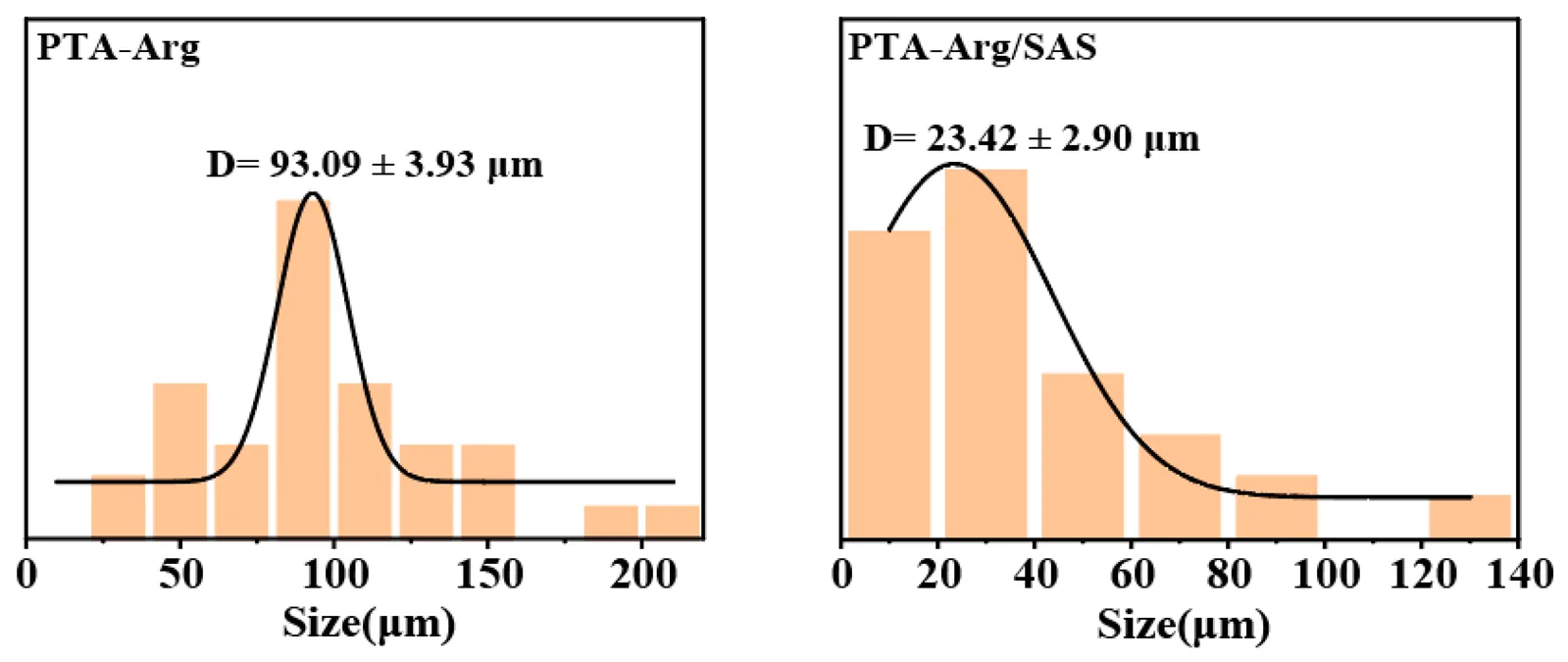
SEM statistical average pore size diagram.

The internal microstructure of hydrogels dictated their fluid absorption capacity, which was a key parameter for wound management materials. The microscopic morphology of the as-prepared hydrogels was characterized via scanning electron microscopy (SEM). As observed in Figure 3a, both the PTA-Arg and PTA-Arg/SAS hydrogels exhibit interconnected three-dimensional network structures. As shown in Figure A1, the PTA-Arg/SAS hydrogel exhibits a smaller and more uniform pore size, with an average diameter of 23.42 μm, whereas PTA-Arg shows a significantly larger average pore size of 93.09 μm. The narrow distribution of PTA-Arg/SAS indicated a homogeneous porous network. This smaller and more evenly distributed pore architecture is advantageous for water absorption and swelling [5]. Moreover, the uniform pores likely contribute to improved structural stability during swelling, minimizing localized stress and potential network collapse. Additionally, the higher effective surface area associated with small, uniform pores may enhance water retention [32]. Furthermore, the porosity of the hydrogels increased significantly with increasing SAS contents (Figure 3b). The porosities of the PTA-Arg, PTA-Arg/SAS1, PTA-Arg/SAS3, and PTA-Arg/SAS5 hydrogels reached 4.14%, 18.83%, 39.41%, and 47.58%, respectively. Covalent bonds and hydrogen bonding between SAS and the PTA network drove the rise in porosity. These interactions jointly promoted the formation of a robust crosslinked network, which effectively prevented structural collapse during the freeze-drying process [41]. Therefore, the morphology and porosity of the hydrogels could be tailored by adjusting the SAS content.

### 3.4. Moisturizing and In Vitro Degradation Performance of PTA-Arg/SAS

**Figure A2:**
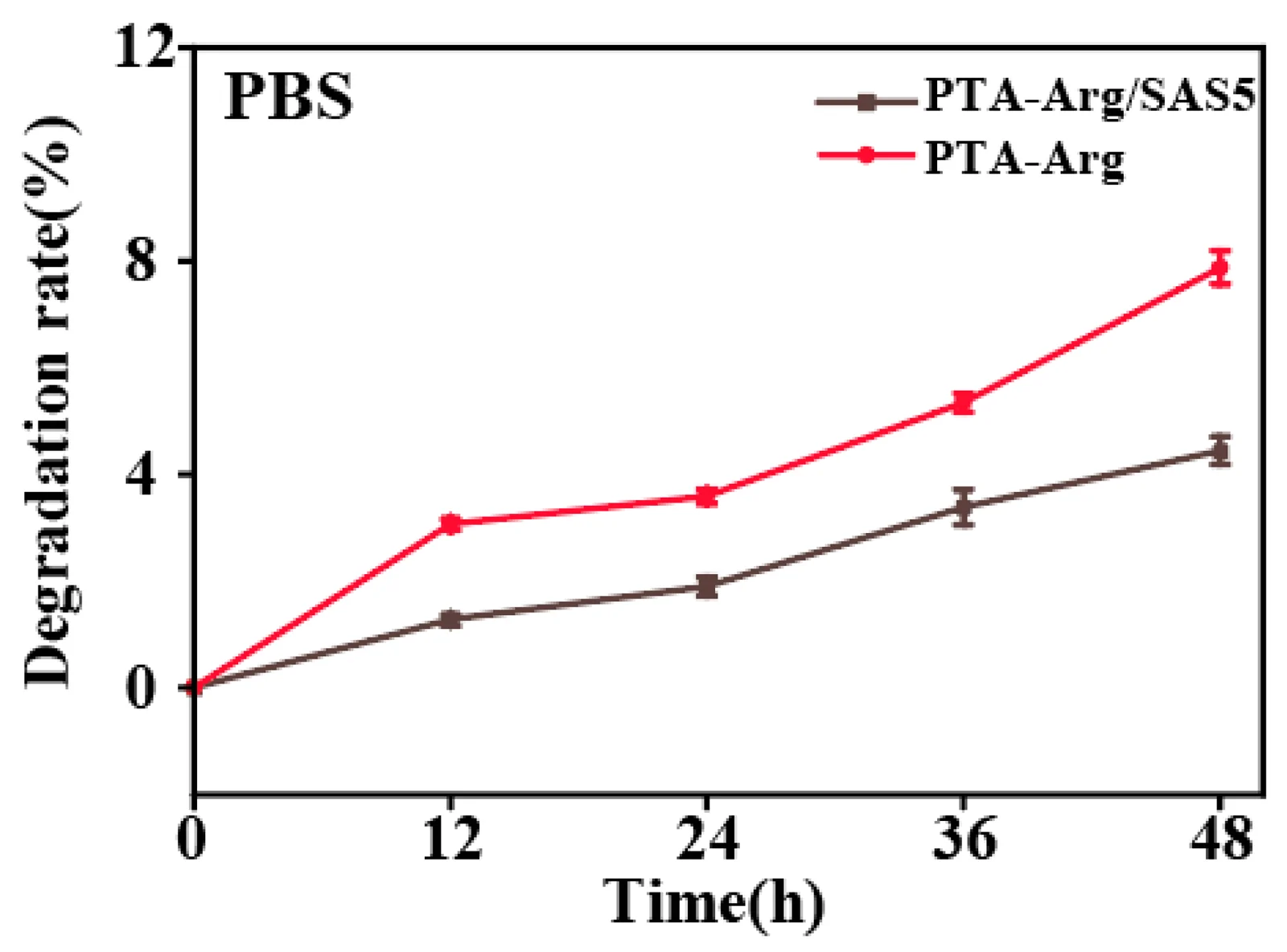
Degradation curve of hydrogels in PBS.

Hydrogel dressings should provide a sustained moisturizing barrier to promote tissue repair [2]. The water retention capacity of the hydrogels is illustrated in Figure 3c. After 84 h, the water retention rates for the PTA-Arg, PTA-Arg/SAS1, PTA-Arg/SAS3, and PTA-Arg/SAS5 hydrogels were recorded at 48.90%, 56.54%, 63.31%, and 73.02%, respectively. Hydrogen bonding between SAS (-OH and -NH_2_) and water molecules stabilized the hydration layer, leading to superior moisturizing ability. Figure 3d shows that all hydrogels are highly hydrated (>80%), particularly the PTA-Arg (86.59%). The equilibrium swelling ratios of the PTA-Arg, PTA-Arg/SAS1, PTA-Arg/SAS3, and PTA-Arg/SAS5 hydrogels reached 118.55%, 128.03%, 140.64%, and 152.29%, respectively (Figure 3e). The improved swelling of PTA-Arg/SAS correlated with their porous structures. The favorable water retention and swelling properties were conducive to maintaining a hydrated environment for tissue repair [23]. At 48 h, the in vitro degradation rates for PTA-Arg and PTA-Arg/SAS5 were 7.87% and 4.45%, respectively (Figure A2). The difference in degradation rates may be due to the hydrogen and S-Ar bonds connecting the SAS and PTA networks. These interactions contributed to the enhanced stability of the hydrogel, thereby reducing its degradation rate.

### 3.5. Rheology and Self-Healing Properties of PTA-Arg/SAS

**Figure 4:**
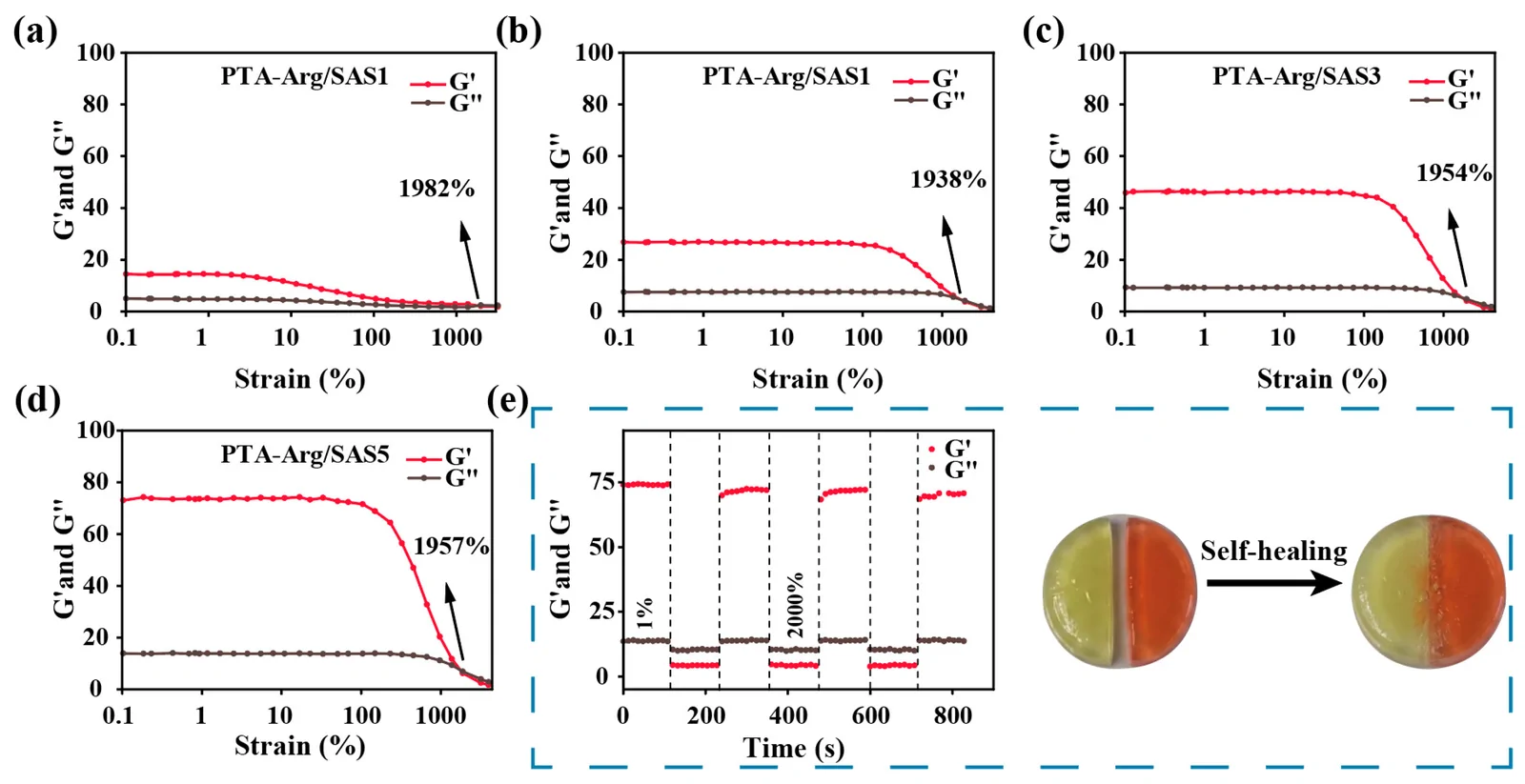
(**a**–**d**) Strain sweep tests of PTA-Arg, PTA-Arg/SAS1, PTA-Arg/SAS3, and PTA-Arg/SAS5. (**e**) Alternating oscillatory strain sweep at 1% and 2000% strain, and macroscopic self-healing process of PTA-Arg/SAS5 hydrogel.

**Figure A3:**
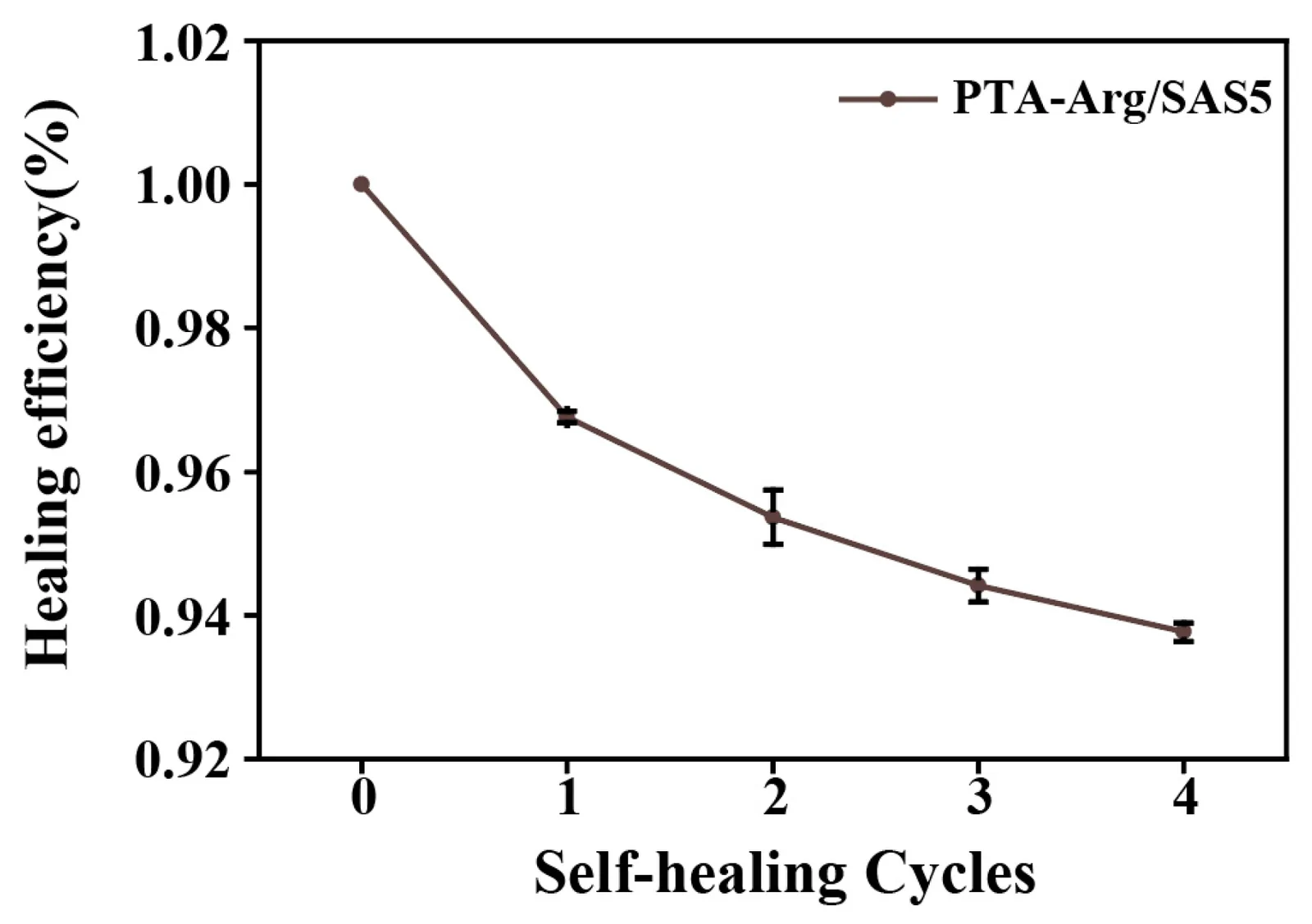
Healing efficiency of hydrogel.

The rheological behaviors of the as-prepared hydrogels were systematically characterized. Mechanical performances were further investigated by recording the storage modulus (G′). Figure 4a–d show that the G′ of PTA-Arg, PTA-Arg/SAS1, PTA-Arg/SAS3, and PTA-Arg/SAS5 hydrogels exceeded the loss modulus (G″) under low-strain conditions. When the strain exceeded 1957%, PTA-Arg/SAS5 underwent a gel-to-sol transition, demonstrating the typical network structure of the hydrogel. With increasing SAS content, the G′ value significantly rose from 14.49 Pa (pure PTA-Arg) to 73.86 Pa (PTA-Arg/SAS5). S-Ar covalent bonds and hydrogen-bonding interactions stabilized the dense SAS/PTA crosslinked architecture [42]. The step-strain measurement in Figure 4e was used to evaluate the recovery behavior of the network. Subjecting the hydrogel to a 2000% strain caused network disruption (G′ < G″). Subsequent strain reduction to 1% fully restored the initial mechanical moduli. The hydrogel showed stable recovery over three consecutive cycles. Moreover, macroscopic experiments provided additional evidence of self-healing properties. When two hydrogel pieces of different colors were brought into contact, they integrated into a single whole. PTA-Arg/SAS exhibits good self-healing properties. To further quantify the self-healing capability, the healing efficiency of the PTA-Arg/SAS5 hydrogel across multiple damage–recovery cycles was calculated based on the rheological G′ values (Figure A3). The healing efficiency was estimated to be approximately 96.76% after the first cycle. As the number of testing cycles increased to four, the efficiency exhibited a slight and gradual decline, eventually reaching approximately 93.77%. This minor decrease could potentially be attributed to the accumulation of a small number of irreversible topological entanglements or incomplete network reconstructions during repeated intense shearing [37].

### 3.6. Adhesive Properties of PTA-Arg/SAS

**Figure 5:**
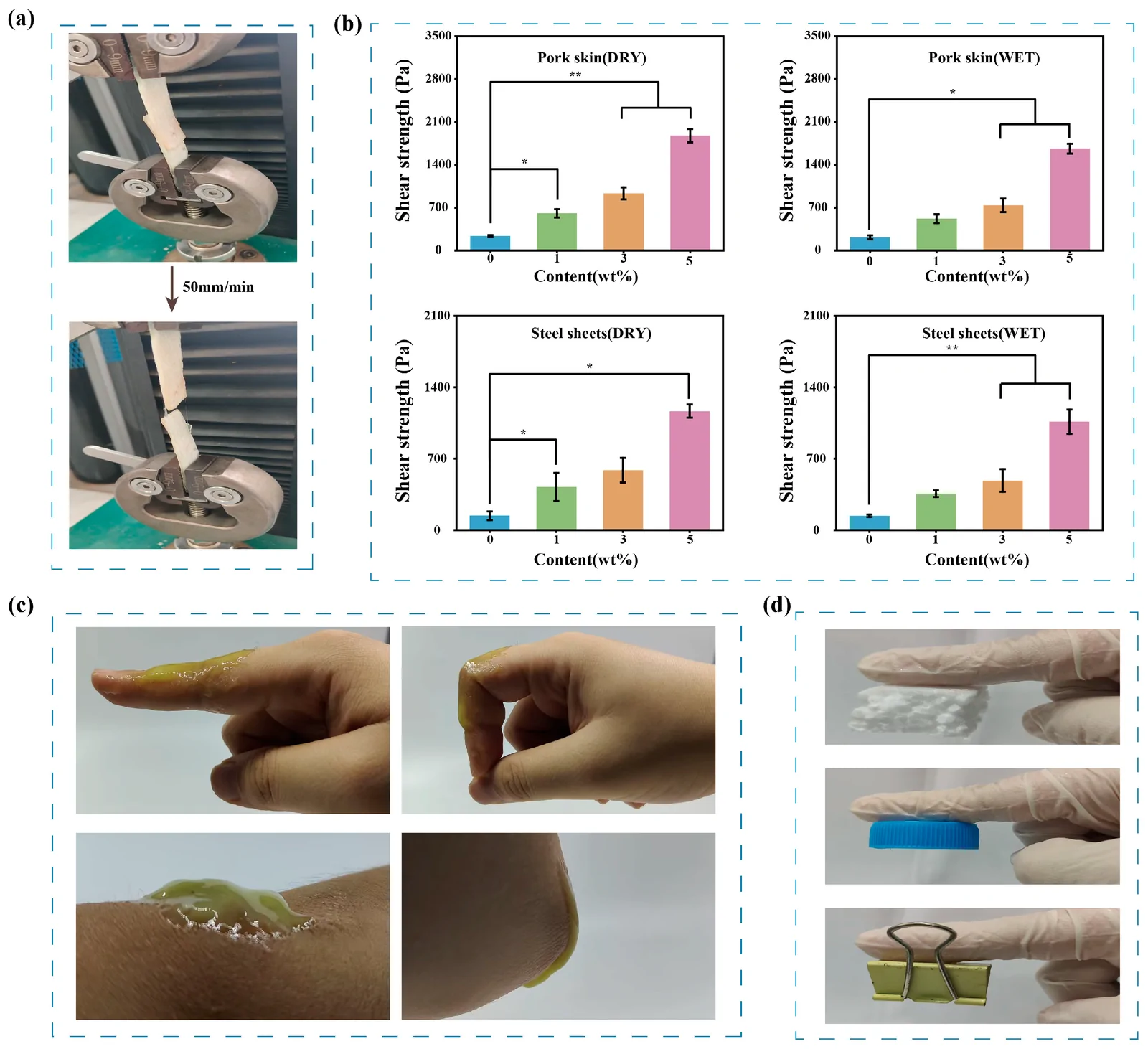
(**a**) The lap-shear test. (**b**) Lap-shear strength of PTA-Arg/SAS hydrogels with different SAS contents toward pork skin and steel sheet under dry and wet conditions (n = 3, ** *p* < 0.01 and * *p* < 0.05). (**c**) Adhesion compatibility of PTA-Arg/SAS5 hydrogel attached to finger and elbow joints under stretching and bending states. (**d**) Universal adhesion demonstration of PTA-Arg/SAS5 hydrogel to various materials such as sponge, plastic bottle cap, and metal clip.

Figure 5a shows the lap-shear tests. Figure 5b displays the shear strength of hydrogels with different SAS contents towards biological tissue (porcine skin, which was bought from the local store, was used in this study) and inorganic substrate (steel sheet) under dry and wet conditions. The baseline PTA-Arg hydrogel exhibited the lowest adhesive strength. Introducing SAS into the system led to a continuous improvement in adhesive performance. The shear strength of PTA-Arg/SAS5 reached 1877 Pa on dry porcine skin and 1166 Pa on dry steel sheets. Even in wet environments, PTA-Arg/SAS5 still maintained favorable adhesive strength, with values of 1663 Pa on wet pork skin and 1064 Pa on wet steel sheets. PTA-Arg/SAS5 formed hydrogen bonds with skin tissues via the -OH and -COOH functional groups [13]. Incorporating SAS elevated the dynamic crosslinking density of the network. This denser structure strengthened internal cohesion, ultimately leading to better adhesion [43]. The conformability test in Figure 5c shows that when the PTA-Arg/SAS5 hydrogel was attached to human fingers and elbow joints, it maintained close contact with the skin without slipping or detachment, even during repetitive bending and stretching. The results demonstrated favorable tissue compliance and interfacial stability. Furthermore, the hydrogel exhibited broad-spectrum adhesion (Figure 5d), attaching to various substrates, including porous sponges, smooth plastic surfaces, and metal clips. Consequently, the hydrogel displayed reliable wet interfacial adhesion and structural stability, which were suitable for applications requiring secure skin attachment.

### 3.7. Injectability and Antioxidant Properties of PTA-Arg/SAS

**Figure 6:**
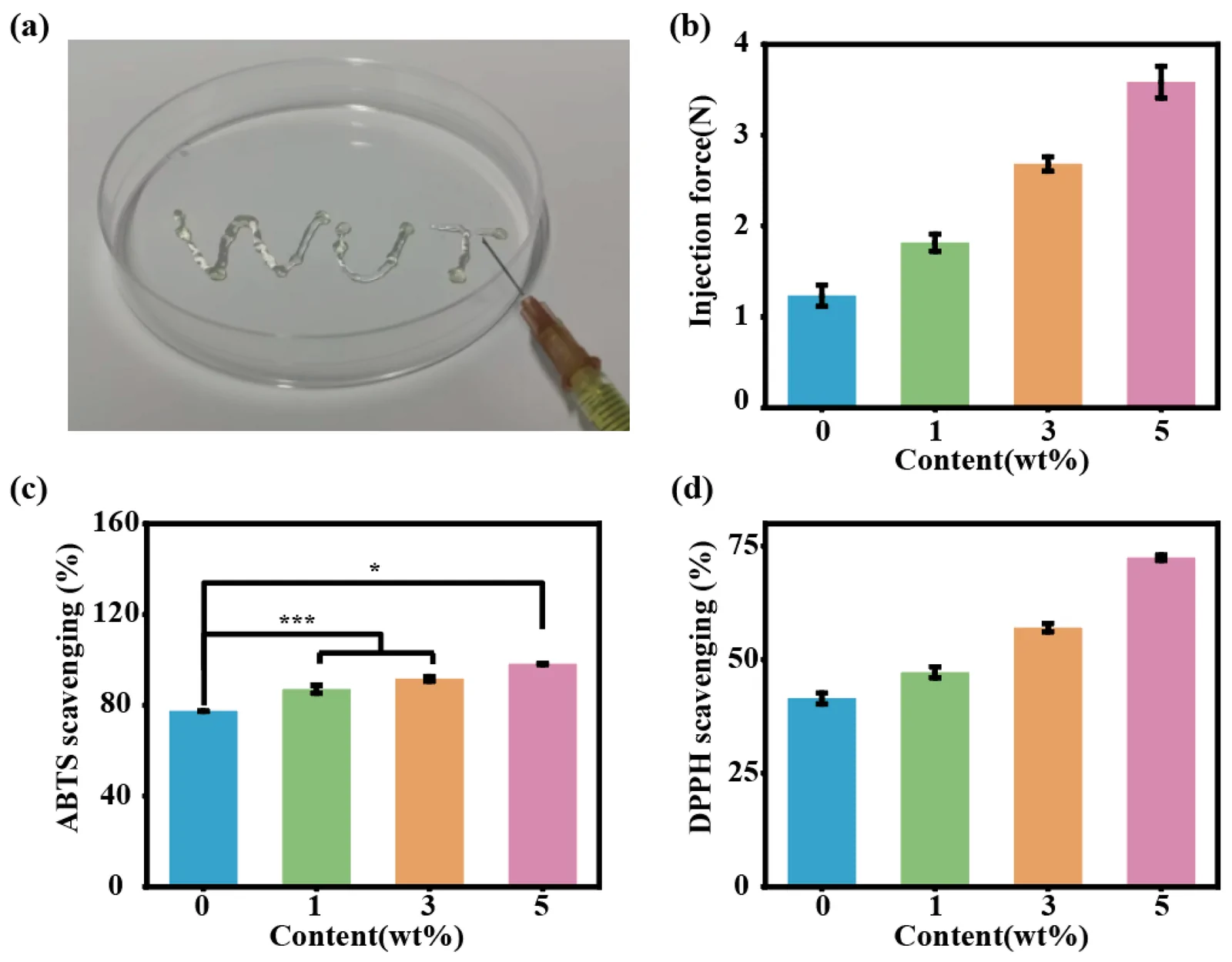
(**a**) Injection and writing performance of PTA-Arg/SAS5 hydrogel through a 21G needle. (**b**) Injection force of SAS-varied hydrogels (21G needle, 50 mm/min). (**c**,**d**) Free radical scavenging tests (ABTS and DPPH) of hydrogels with different SAS contents (n = 3, *** *p* < 0.001, and * *p* < 0.05).

Successful extrusion through a 21G needle to spell “WUT” confirmed the injectability of the PTA-Arg/SAS5 (Figure 6a). As the SAS content increased from 0 to 5 wt%, the injection force progressively increased from approximately 1.2 N to 3.5 N (Figure 6b). The peak injection force of PTA-Arg/SAS5 stayed markedly lower than the threshold for routine manual injection documented in the previous literature, supporting its practical usability [25,44]. High localized concentrations of ROS impaired the healing process. Therefore, evaluating the in vitro antioxidant performance of the hydrogels was essential. Figure 6c,d displays the ABTS and DPPH radical scavenging efficiencies of the prepared hydrogels, respectively. The PTA-Arg hydrogel exhibited intrinsic antioxidant activity, with an ABTS scavenging rate of 77.4% and a DPPH scavenging rate of 41.5%. This capability was attributed to the reducing nature of the sulfur-containing groups within the PTA network. Increasing the SAS concentration improved the antioxidant properties of the hydrogels. Tests on the PTA-Arg/SAS5 formulation demonstrated a 98.2% ABTS clearance alongside a 72.7% reduction in DPPH. This improved antioxidant performance was attributed to the active amino acid residues in the Ser backbone and the phenolic hydroxyl groups of the grafted SA, which can effectively quench free radicals in the microenvironment [45,46]. Accordingly, the demonstrated in vitro antioxidant properties were conducive to mitigating oxidative stress in the local microenvironment.

### 3.8. Antibacterial Properties of PTA-Arg/SAS

**Figure 7:**
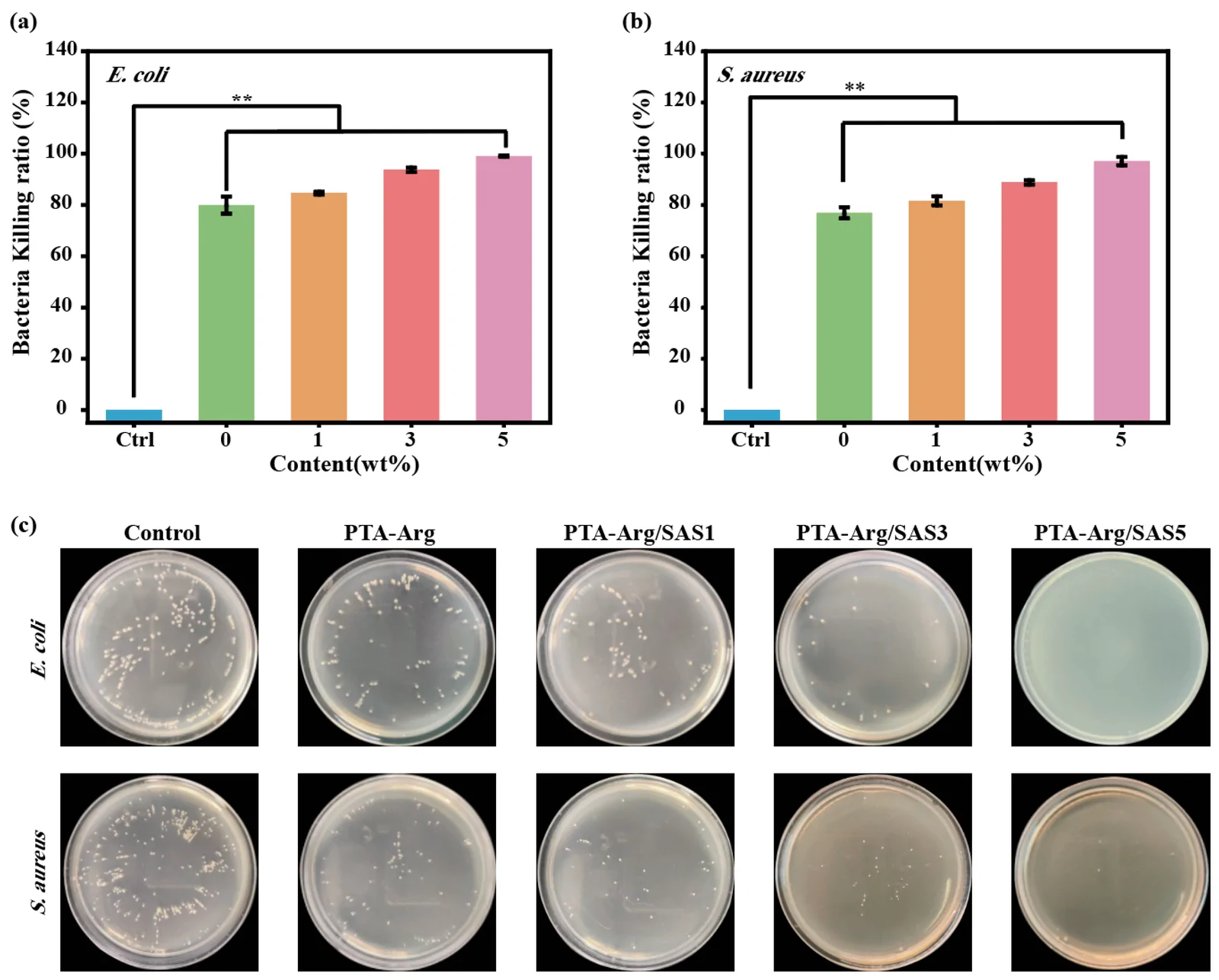
(**a**,**b**) Antibacterial performance of hydrogels with different SAS contents against *E. coli* and *S. aureus* (n = 3, ** *p* < 0.01). (**c**) Agar plates treated with various hydrogels.

The in vitro plate spreading assays revealed that the PTA-Arg killed 79.9% of *E. coli* and 76.8% of *S. aureus* (Figure 7a,b). The antibacterial efficacy demonstrated a concentration-dependent behavior with rising SAS content. At 5 wt% SAS, the PTA-Arg/SAS5 eliminated 99.1% of *E. coli* and 97.0% of *S. aureus*. Furthermore, the photographs of the bacterial culture plates (Figure 7c) visually corroborate these results. The demonstration of antibacterial activity by the hydrogels was primarily attributed to the physicochemical effects of specific functional groups within the network. The cationic guanidinium groups of Arg electrostatically bind to anionic bacterial membranes [18]. Such interactions may perturb membrane integrity, increase membrane permeability, and eventually contribute to bacterial membrane disruption [47]. Furthermore, SA may disturb bacterial envelope integrity and increase membrane permeability, resulting in the leakage of intracellular components such as nucleic acids and proteins [48]. SA may also disrupt membrane polarization, thereby interfering with bacterial energy metabolism and normal physiological processes [49,50,51]. Therefore, the enhanced antibacterial performance with increasing SAS content may be attributed to the cooperative effects of Arg and SA. These effects contributed to the favorable antibacterial performance of the PTA-Arg/SAS hydrogel.

### 3.9. Biocompatibility of PTA-Arg/SAS

**Figure 8:**
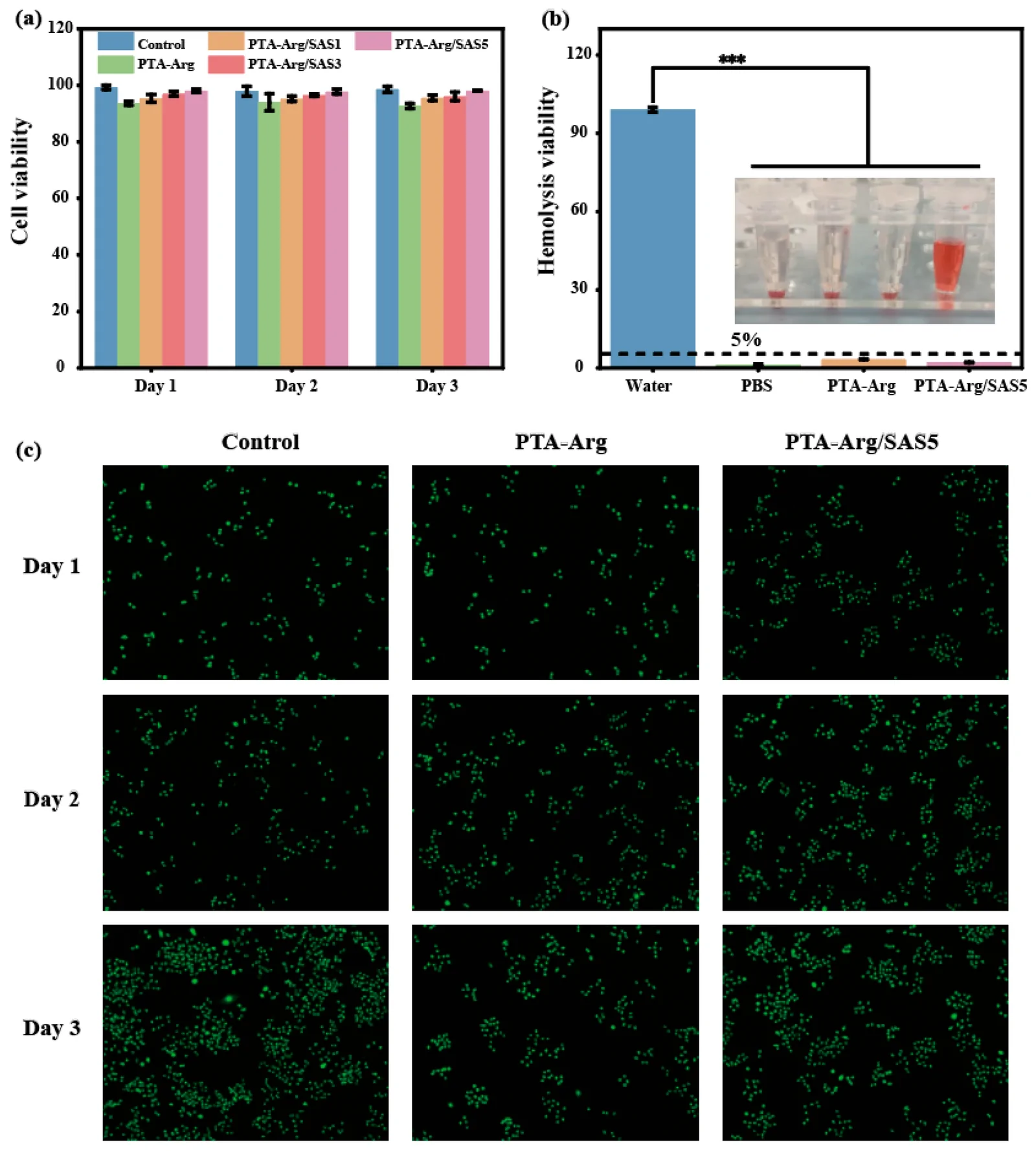
(**a**) Viability evaluation of L929 cells. (**b**) Determination results of the hemolysis rate (n = 3, *** *p* < 0.001). (**c**) Live/dead staining of hydrogel-treated cells.

Good biocompatibility is an essential requirement for biomaterials intended for tissue contact [17,36]. Cell viability and hemocompatibility across all SAS concentrations were assessed using live/dead staining, CCK-8 assays, and hemolysis tests. L929 cell viability consistently exceeded 90% across all groups over a 3-day incubation period (Figure 8a). Corresponding fluorescence micrographs corroborated the favorable cellular status (Figure 8c). Fluorescence imaging over three days revealed predominantly viable (green) cells alongside minimal dead (red) signals. Concurrently, the cell density progressively increased over the culture duration. Overall, the hydrogels were highly cytocompatible. Furthermore, Figure 8b demonstrates that the hemolysis ratios of the PTA-Arg and PTA-Arg/SAS5 hydrogels were negligible, both falling well below the 5% safety threshold specified by the International Organization for Standardization [52,53]. As shown in the inset photographs, the positive control group exhibited a uniform red color, whereas the supernatants of the hydrogel groups remained transparent, which was consistent with the PBS negative control group.

## 4. Conclusions

In summary, the multifunctional PTA-Arg/SAS composite hydrogel was successfully fabricated, featuring robust tissue adhesion, potent antibacterial activity, and favorable antioxidant capacity. Arginine serves a dual purpose: it initiates the aqueous ring-opening polymerization of thioctic acid (TA) to form the PTA network, while its cationic guanidinium groups endow the system with intrinsic antibacterial properties. Furthermore, the incorporation of salicylic acid-grafted sericin (SAS) significantly enhances the structural stability of the hydrogel. SAS effectively suppresses PTA depolymerization through the formation of stable S-Ar bonds and dynamic hydrogen-bonded networks. Concurrently, the salicylic acid moieties on the SAS side chains synergistically amplify the overall antioxidant and bactericidal performance. Combined with its excellent in vitro biocompatibility, this structurally stable and bioactive hydrogel provides a promising platform for advanced tissue-interfacing engineering and complex wound therapy.

## Data Availability

The raw data supporting the conclusions of this article will be made available by the authors on request.
