# Correction: Yuan et al. Multifunctional Poly(thioctic acid) Composite Hydrogels with Self-Healing, Antibacterial, Antioxidant, and Adhesive Properties. *Materials* 2026, *19*, 2695

**DOI:** 10.3390/ma19153273

**Published:** 2026-08-03

**Authors:** Yang Yuan, Jiawei Zhang, Fangzheng Yu, Chen Wang, Jiale He, Zheng Zhao

**Affiliations:** 1 Sanya Science and Education Innovation Park, Wuhan University of Technology, Sanya 572000, China; 348318@whut.edu.cn (Y.Y.); zjw9801@126.com (J.Z.); 348317@whut.edu.cn (F.Y.); wangchen__123@163.com (C.W.); hjl2024305118@163.com (J.H.); 2 State Key Laboratory of Advanced Technology for Materials Synthesis and Processing, Wuhan University of Technology, Wuhan 430070, China

In the original publication [1], the funder “Hainan Provincial Sanya Yazhou Bay Science and Technology Innovation Joint Project (No. ZDYF2025GXJS141) and Wuhan Metropolitan Area Collaborative Technology Innovation Project (No. 2025070604020039)” was not included.

The authors state that the scientific conclusions are unaffected. This correction was approved by the Academic Editor. The original publication has also been updated.
